# Reply: Targeted Therapy for Hypertension in Pregnancy: Hemodynamics as a Therapeutic Compass

**DOI:** 10.1016/j.jacadv.2025.102521

**Published:** 2026-01-19

**Authors:** Stephanie A. Leonard, Elliott K. Main, Mark A. Hlatky, Krista F. Huybrechts, Brian T. Bateman

**Affiliations:** a Stanford University School of Medicine, Stanford, California, USA; b Brigham and Women’s Hospital, Harvard Medical School, Boston, Massachusetts, USA

We thank Dr Ceulemans and colleagues for their letter in response to our study,^1^ in which we found evidence to support the conclusion that labetalol and nifedipine have similar effectiveness and safety in treating chronic hypertension during pregnancy. Dr Ceulemans and colleagues bring attention to European studies on the use of transthoracic echocardiography to measure cardiac output and peripheral vascular resistance to identify hemodynamic subtypes among pregnant individuals and direct medication treatment decisions.^2^^,^^3^ These studies represent an active area of research and are of interest. To our knowledge, the use of hemodynamic subtypes in pregnancy is not used outside of research settings in the United States. The ACOG (American College of Obstetricians and Gynecologists) recommends both labetalol and nifedipine as first-line treatments, and the results of our study and others suggest that the medications are used interchangeably in practice.^1^^,^^4^ However, ACOG does recommend that nifedipine be avoided in individuals with tachycardia and that labetalol be avoided in individuals with asthma, preexisting myocardial disease, decompensated cardiac functions, heart block, and bradycardia.^4^ These contraindications may have relationships to hemodynamic subtypes.

Additionally, Dr Ceulemans and colleagues correctly note that our study population consisted of individuals with chronic hypertension and therefore our results may not apply to individuals with preeclampsia. Research on hemodynamic subtypes has focused on the prevention and treatment of preeclampsia, with very limited inclusion of individuals with chronic hypertension.^2^^,^^3^ Hemodynamics may differ between individuals who start pregnancy with chronic hypertension and those who do not. Although we did not have data on measurements of hemodynamic parameters during pregnancy, this would be an important area of future research.

Finally, Dr Ceulemans and colleagues question whether differences in low-dose aspirin use between treatment groups may have influenced the study results. We believe the likelihood of confounding by low-dose aspirin use is low because prescription aspirin dispensing (inclusive of low-dose aspirin) was similar between the treatment groups as were other measured characteristics, including those indicative of more severe hypertension. Furthermore, ACOG recommends low-dose aspirin use for all pregnant individuals with chronic hypertension to help prevent or delay preeclampsia.^4^^,^^5^
